# HCV infected prisoners: should they be still considered a difficult to treat population?

**DOI:** 10.1186/1471-2334-13-374

**Published:** 2013-08-14

**Authors:** Fabio Iacomi, Giuseppina Iannicelli, Andrea Franceschini, Paolo Migliorisi, Silvia Rosati, Pierluca Piselli, Paola Scognamiglio, Gabriella De Carli, Sonia Marcellini, Fabrizio Palmieri

**Affiliations:** 1 Clinical Department, National Institute for Infectious Diseases, L. Spallanzani, Rome, Italy; 2 Unit of Penitentiary Medicine ASL RM A, Rome, Italy; 3 Epidemiology Department, National Institute for Infectious Diseases, L. Spallanzani, Rome, Italy; 4 Unit of Penitentiary Medicine ASL RM B, Rome, Italy; 5 Divisione Post-Acuzie, Via Portuense, 292-00149, Rome, Italy

**Keywords:** Hepatitis C, Correctional facility, Inmates, Sustained response

## Abstract

**Background:**

The prevalence of chronic hepatitis C virus (HCV) infection in the Italian correctional population is estimated to be around 38%. In this setting HCV infection treatment is controversial because of several factors such as active drug substance abuse, psychiatric illness, length of treatment, risk of re-infection, poor adherence and low success rate.

**Methods:**

A retrospective data review of 159 inmates, positive for anti-Hepatitis C virus (HCV) antibody, evaluated to National Institute for Infectious Diseases “L. Spallanzani” (INMI) from January 2006 to December 2009, was conducted to evaluate rate of completion (feasibility) and outcome efficacy of chronic Hepatitis C Virus (HCV) infection treatment with Pegylated Interferon and Ribavirin in five correctional facilities in Rome.

**Results:**

Of the 159 inmates evaluated in the study period, 50, all male (median age 39 years) were treated. Twenty patients (40%) did not complete treatment: 15 showed no response and therapy was stopped, 5 patients (10%) interrupted treatment because of adverse reactions. The global feasibility was 60%. The overall sustained virologic response (SVR) was 50% (32% for genotype 1 and 68% for genotype other than 1). The main predictors of SVR at the Multivariable Logistic Regression Odds Ratio (MLR-OR) were a better pretreatment histological diagnosis (absence of bridging fibrosis or cirrhosis [MLR-OR 11.85; 95% CI 1.96-71.62) and a HCV genotype other than 1 (MLR-OR 5.87; 95% CI 1.49-23.17).

**Conclusions:**

Chronic HCV infection treatment in correctional facilities is feasible and effective and should be strongly recommended, in combination with preventive measures, in appropriately screened patients because it represents an important opportunity to treat a population with a high prevalence of chronic HCV infection among whom treatment options post incarceration may be limited.

## Background

In Italy the estimated prevalence of anti-Hepatitis C virus (HCV) antibody seropositivity in the general population is 2,9%,with a north–south gradient and increasing with age [1,2]. Rates are considerably higher in the Italian correctional population (38%) because of the higher proportion of intravenous drug users (IVDUs) [3].

Despite the relatively high success rates reported in the U.S. and Canada correctional population [4-9], several factors reported as potential obstacles to treatment of chronic HCV infection in the general population, such as active drug substance abuse, psychiatric illness, length of treatment, risk of re-infection, poor adherence and low success rates, may be more prevalent in this setting [5,8,10].

Many accurate data are published on the prevalence of HCV infection in the correctional population in Europe [2,11,12], but in the same population few data are available on the outcome of treatment of chronic HCV infection [12,13].

To evaluate feasibility and efficacy of treatment of chronic HCV infection in this setting, a retrospective review of medical records was performed in a cohort of inmates in five correctional facilities in Rome.

## Methods

### Patients

Were retrospectively evaluated data of 159 inmates (148 males, 11 females) who tested positive for anti-HCV antibody (HCV-Ab) at their entry in five correctional facilities in Rome (Casa Circondariale(CC) Regina Coeli, and Istituti Penitenziari Rebibbia, which include: CC Nuovo Complesso, CC Femminile, Casa di Reclusione, III Casa, Casa di Reclusione; average daily census 2541 in the study period) and were sent for consultation at the National Institute for Infectious Diseases “L. Spallanzani” (INMI), Rome, from January 2006 to December 2009.

All inmates were tested for HCV-Ab, HCV viremia (HCV-RNA), human immudeficiency virus antibodies (HIV-Ab) and hepatitis B surface antigen (HBsAg). Serologic tests were performed using microparticle enzyme immunoassays (EIAs) for HBsAg (AxSYM, Abbott, Wiesbaden, Germany), HCV 3.0 third-generation EIAs (Abbott) for HCV-Ab and the Genscreen HIV 1/2 ELISA (BioRad, Marnes La Coquette, France) for HIV-Ab. HCV-RNA was measured using the COBAS Taq-Man HCV test (Roche Molecular System) with a detection limit of 12 IU/ml. If patients had HCV-RNA detectable in serum, HCV genotype was determined using the reverse hybridization method (InnoLipa HCV II; Siemens Medical Solutions Diagnostics, Tarrytown, NY), those with an expected length of stay in the correctional facility of less than 12 (for genotypes 2, 3) or 18 (for genotypes 1, 4) months necessary for evaluation, uninterrupted treatment and follow-up were not considered eligible for treatment. The remaining population underwent clinical and laboratory evaluation to assess contraindications to treatment with interferon and ribavirin, including psychiatric consultation and screening for drugs or alcohol abuse: patients were considered eligible for immediate treatment if they were on rehabilitation or stable maintenance agonist therapy (methadone) according recommendations of Italian Association for the Study of the Liver (A.I.S.F.), Italian Society of Infectious and Tropical Diseases (S.I.M.I.T.), Italian Federation Department’s Operators and Addiction Services (FederSerD), Italian Prison Medicine and Healthcare Society (S.I.M.S.Pe) [14,15].

For the many inmates who were in the process of being transferred to other correctional facilities depending for health assistance from other institutions outside Rome, or were going to be released and living outside our area, initiation of treatment was deferred and they were referred for treatment and clinical and virological follow-up to other healthcare facilities in the place of final residence.

Information on length of incarceration was available on clinical charts only as categorical variable: for genotypes 1 and 4 < or > of 18 months; for the other genotypes < or> of 12 months.

**Figure 1:**
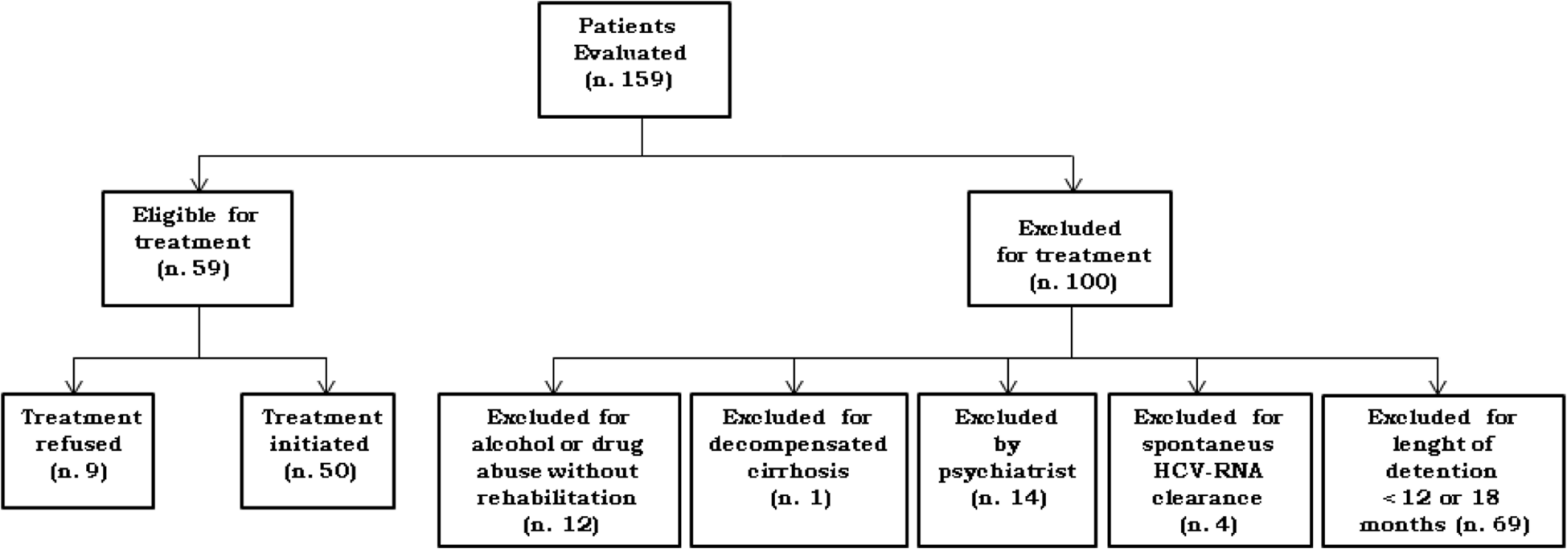
Algorithm for evaluation of patients eligible to the treatment.

Figure 1 shows the decisional algorithm for eligibility to treatment.

Patients were offered treatment if they had undergone a liver biopsy at INMI that was consistent with chronic hepatitis and had been categorized as F1 to F4 according to METAVIR system for fibrosis staging [16].

Standard guidelines for treatment of chronic HCV infection, available at the time of patient’s evaluation, were followed [17]. Genotypes 1 and 4 were treated for 48 weeks with Pegylated Interferon-α2a, 180 μcg subcutaneously once a week, in combination with Ribavirin 15 mg/kg/day. Genotypes 2 and 3 were treated for 24 weeks with Pegylated Interferon-α2a, 180 μg subcutaneously once a week in combination with Ribavirin 800 mg/day.

Pegylated Interferon-α2a was administered by directly observed therapy (DOT) while Ribavirin was self administered.

Side effects were regularly monitored and therapy was modified or stopped according to standard guidelines.

In accordance with provisions of the regulatory authority “Agenzia Italiana del Farmaco” (A.I.F.A.) in force at 2008, when we had conducted this study, the approval of the Ethics Committee was not required for retrospective observational studies [18].

### Data analysis

The measure of feasibility was the rate of treatment completion. The measure of efficacy was the rate of sustained virologic response (SVR), defined as undetectable HCV-RNA in serum at the end of follow-up, 24 weeks after treatment withdrawal. The whole treated population - i.e. all patients who received at least one dose of study medication- was included in the analysis (intention to treat analysis).

Association between SVR and selected patients’ characteristics was assessed by means of Odds Ratios (ORs) and their 95% Confidence Intervals (95% CI) in order to define predictors of SVR in the study population using Logistic Regression.

χ^2^ test (or Fisher’s exact test when applicable) or Mann Whitney non-parametric test were used to compare groups for categorical or continuous variables, respectively.

Univariable analysis was conducted to select significant variables (p<0.10) to be included in the multivariable analysis, in which Multivariable Logistic Regression Odds ratio (MLR-OR) was calculated. Were considered two different models: Model I in which all selected variables were included, and Model II in which the final model included only those variables selected after a backward elimination (p<0.10) of those variables included in Model I.

Statistical analysis was performed using SPSS ver. 19 (SPSS Inc).

## Results and discussion

Fifty-nine out of 159 (37%) inmates evaluated in the study period were considered eligible for treatment; only nine declined therapy while 50 patients started treatment. No difference in the baseline characteristics was found between treated subjects and those who refused therapy. The overall HCV treatment rate was 31.4% (50/159), while 100 patients were considered ineligible for treatment and nine declined therapy. No difference in the baseline characteristics was found between treated subjects and those who refused therapy.

Among 100 patients considered ineligible, the most frequent reason (69%) for not-treatment was length of detention. As explained in methods section, we could not calculate the median length of incarceration of these patients, but this information was available for overall inmates in the five correctional facilities (source: “Department of Penitentiary Administration - Ministry of Justice”). The overall average length of incarceration in the five correctional facilities during the study was 9.5 months . In 26 patients treatment was contraindicated for psychiatric problems (14 patients) or for substance abuse (12 patients). For the remaining 5% treatment was not indicated due to absence of HCV replication (4 patients) or decompensated cirrhosis (1 patient).

All 50 treated patients underwent liver biopsy. Among them 34 (68%) had a substance abuse history and 47 (94%) were naïve for interferon and ribavirin.

Baseline characteristics of eligible patients are summarized in Table 1.

**Table 1:** Baseline characteristics of 50 inmates treated for chronic HCV infection at national institute for infectious diseases L. Spallanzani, 2006-2009

**Characteristics**	**Treated**	
	**N**	**%**
All	50	
**Age**		
Median (IQR)	39 (34–43)	
<35	15	30.0
35-44	25	50.0
≥45	10	20.0
**Race**		
Caucasian	46	92.0
African	4	8.0
BMI		
<25	24	48.0
≥25	26	52.0
**IVDU**		
Yes	34	68.0
No	16	32.0
**Biopsy staging (Metavir)**		
F1-F2	37	74.0
F3-F4	13	26.0
**ALT (IU/L)**		
<60	10	20.0
≥60	40	80.0
**HCV-RNA (mU/ml)**		
<6x10^5^	18	36.0
≥6x10^5^	32	64.0
**HCV genotype**		
1	25	50.0
Other than 1	25	50.0
**HIV Ab**	3	6.0
Positive	47	94.0
Negative		

Thirty patients (60%), 11 with genotype 1, one with genotype 4, 17 with genotype 3 (one co-infected with HIV) and one with genotype 2, showed an end of treatment response: 25 (83%) achieved a SVR, including the HIV co-infected patient, while 5 (17%) relapsed. The global SVR rate was 50%. SVR according to genotype was 32% (8/25) for genotype 1, 50% (1/2) for genotype 4 and 70% (16/23) for genotypes 2–3.

Twenty patients did not complete treatment: 15 showed no response (9 null and 6 non responder) and therapy was therefore stopped, including two HIV co-infected patients with genotype 1, while 5 patients interrupted treatment because of adverse reactions (two cases of severe thrombocytopenia, and one each of interstitial pneumonia, severe rash and depression).

Univariable regression analysis showed that younger age (<40 years, OR=3.27, p=0.048), better pretreatment histological diagnosis (F1-F2 vs. F3-F4, OR=9.04, p=0.009), HCV genotype other than 1 (OR=4.52, p=0.013) and lower baseline viral load (<6×10^5^ UI/mL, OR=2.92, p=0.082) were predictive of SVR (Table 2).

**Table 2:** Univariate analysis of the association of SVR with patients characteristics

		**SVR**		**No SVR**		**Tot**	**OR**	**95% CI**	**p**
		**N**	**%**	**N**	**%**				
Age (years)									
	40+	7	33.3	14	66.7	21	1		
	<40	18	62.1	11	37.9	29	3.27	1.01-10.62	0.048
BMI (kg/m^2^)									
	<25	10	41.7	14	58.3	24	1		
	25+	15	57.7	11	42.3	26	1.91	0.62-5.88	0.260
IVDUs									
	Yes	16	47.1	18	52.9	34	1		
	No	9	56.3	7	43.8	16	1.45	0.44-4.78	0.545
Biopsy staging (Metavir)									
	F3-F4	2	15.4	11	84.6	13	1		
	F1-F2	23	62.6	14	37.8	37	9.04	1.74-46.89	0.009
ALT (mU/mL)									
	60+	18	45.0	22	55.0	40	1		
	<60	7	70.0	3	30.0	10	2.85	0.-64-12.64	0.168
HCV genotype									
	1	8	32.0	17	68.0	25	1		
	Not 1	17	68.0	8	32.0	25	4.52	1.38-14.82	0.013
^a^HCV viral load (IU/ml)									
	High	13	40.6	19	59.4	32	1		
	Low	12	66.7	6	33.3	18	2.92	0.87-9.78	0.082

^a^categorized in High (≥6×10^5^ IU/ml) and Low (<6×10^5^ IU/ml).

*BMI* Body Mass Index, *IVDUs* Intravenous drug users, *SVR* Sustained Virologic Response, *OR* Odds Ratio, *CI* Confidence Interval.

Multivariable logistic regression analysis after backward elimination procedure, shown in Table 3, confirmed that better pretreatment histological diagnosis (absence of bridging fibrosis or cirrhosis) and a HCV genotypes other than 1 were best predictive of SVR.

**Table 3:** Multivariate analysis of the association of SVR with selected patients characteristics

		**Model I**		**Model II**	
		**MLR-OR**	**95% CI**	**MLR-OR**	**95% CI**
Age					
	40+	1			
	<40	1.78	0.42-7.47		
Biopsy staging (Metavir)					
	F3-F4	1		1	
	F1-F2	9.29	1.47-58.64	11.85	1.96-71.62
HCV genotype					
	1	1		1	
	Not 1	5.19	1.27-21.18	5.87	1.49-23.17
^a^HCV viral load (IU/ml)					
	High	1			
	Low	1.61	0.38-6.91		

Model I: Including all variables found to be significant (p<0.10) at univariate level.

Model II: Final model obtained through backward elimination starting from all variables included in Model I.

^a^categorized in Low (<6×10^5^ IU/ml) and High (≥6×10^5^ IU/ml).

*SVR* Sustained Virologic Response, *MLR-OR* Multivariable Logistic Regression Odds Ratio, *CI* Confidence Interval.

Few data are available on the outcome of treatment of chronic HCV infection in the correctional population in Italy with no study in patients who all undergone to liver biopsy.

The rate of treatment completion (60%), the proportion of patients that interrupted treatment because of adverse events (10%), the overall rate of SVR (50%) and genotype-specific rates of SVR (32% for genotype 1, and 68% for genotypes other than 1), were substantially similar to those reported in previous and recent studies among American inmates [3-6], and in the community [19-21]. Genotypes other than 1 and absence of bridging fibrosis or cirrhosis were independently associated with SVR, a finding well established in the literature [19,20,22].

Factors reported as potential obstacles to treatment of chronic HCV infection, especially in correctional populations [5,8,10], such as active drug substance abuse, psychiatric illness, length of treatment and poor adherence did not affect the end points of this study.

Feasibility and efficacy of treatment among patients with a previous history of IVDU did not differ significantly from those of patients without an history of IVDU (61% and 47.1% vs 58% and 56.3%, respectively); treatment withdrawal rate caused by psychiatric illness was very low (2%), and finally, because patients were considered eligible for treatment if they had an expected length of stay of at least 12 or18 months in the facility, adequate treatment and follow-up duration were ensured by INMI and no patient was lost to follow-up.

Despite advances in treatment and remarkable improvements in cure rates, few persons with chronic HCV infection are receiving treatment in some settings.

Studies in various correctional facilities have suggested that 59 to 85% of patients presenting with confirmed HCV infection go untreated; [8,10,12,13]. Recently Rice has reported a lower proportion (40%) of untreated patients [9].

In general populations this proportion varies from 73% to 91% [23-26]. In our study population the 68.6% of patients did not receive HCV treatment, consistent with that reported among patients referred to correctional facilities.

The most frequent reason (69%) for non-treatment was the length of detection. Spaulding reports that only a small proportion of inmates are treated: those with an expected remaining stay in prison at least 18 to 24 mounths [27]. We believe that the minimum duration of detention, necessary for uninterrupted treatment and follow-up for patients eligible for an immediate treatment must be at least 12 months for genotypes 2 and 3, and 18 months for genotypes 1 and 4. This issue highlights the importance of postdischarge planning, adequate community resources, and continuity of care for inmates, in whom the potential exists for successful treatment outcomes, in order to increase the access to treatment.Continuity of care for prison inmates with chronic HCV infection is ensured by INMI through outpatients’ service.

Chronic HCV infection can lead to cirrhosis and liver cancer and contributes to morbidity and mortality. Given the high prevalence of chronic HCV infection in the correctional facilities, it is important to identify and evaluate inmates for treatment.

The average length of incarceration in the five correctional facilities is 9.5 months, and most incarcerated individuals return to their communities. Therefore, treatment of chronically infected individuals in the correctional setting may be an effective strategy to reduce the incidence and prevalence of viral hepatitis in the community. Indeed, prisons could serve as a reservoir, resulting in the amplification of HCV and other infectious disease transmission in the community after the release of infected inmates or of those who became infected while incarcerated [28].

In the future new therapies with direct-acting antivirals (DAAs) and evolving standard of care will challenge the exclusion based on length incarceration. Moreover in this population use of new DAAs, currently available, and those available in the future, might markedly improved SVR rates in the treatment of chronic HCV infection [29-32].

Despite the promising scenario of future therapies, HCV infected inmates will remain for many years a hard to reach and hard to treat population. Therefore, public health and correctional institutions should collaborate to develop prevention programs including immunization, health education and substance abuse treatment for inmates. It is worrisome that not all prisoners undergo screening for these infections. Data from a sample of inmates evaluated for bloodborne infections in 2009–2010 demonstrate that 11.7% of inmates were screened for HIV, while HCV and HBV testing percentage was lower [33]. The study has some limitations which may impact our findings. Being a retrospective study, a significant sampling selection bias might have occurred, as anti-HCV screening of inmates does not cover the entire correctional populations. Moreover since we have evaluated only inmates for which the correctional physician requested a specialist consultation at our Institute, a population of patients more suitable to treatment could have been selected. Moreover, all treated subjects were males, which limits the generalizability of the results to the female inmate population.

## Conclusions

In this study treatment of chronic HCV infection in correctional facilities was feasible and safe with good chances to achieve SVR. Also in this population use of new DAAs currently available, and those available in the future, might markedly improved SVR rates in the treatment of chronic HCV infection.

Treatment of chronic HCV infection in correctional facilities should be strongly recommended, in combination with preventive measures, in appropriately screened patients because it represents an important opportunity to treat a population with a high prevalence of chronic HCV infection among whom treatment options post incarceration may be limited.

## Competing interests

The authors declare that they have no competing interests.

## Authors’ contributions

FI designed the study, interpreted the results and wrote the paper and submitted the final version. GI collected the data. AF designed the study, interpreted the results and collected the data. PM collected the data. SR designed the study and collected the data. PP designed the analyses, analyzed the data and interpreted the results. PS designed the analyses, analyzed the data, interpreted the results and reviewed the manuscript. GDC designed the analyses, analyzed the data, interpreted the results and reviewed the manuscript. SM collected the data. FP designed the study, interpreted the results and reviewed the manuscript. All authors read and approve the final manuscript.

## Pre-publication history

The pre-publication history for this paper can be accessed here:

http://www.biomedcentral.com/1471-2334/13/374/prepub
